# A Comprehensive Review of the Global Epidemiology, Clinical Management, Socio-Economic Impacts, and National Responses to Long COVID with Future Research Directions

**DOI:** 10.3390/diagnostics14111168

**Published:** 2024-05-31

**Authors:** Xiufang Song, Weiwei Song, Lizhen Cui, Tim Q. Duong, Rajiv Pandy, Hongdou Liu, Qun Zhou, Jiayao Sun, Yanli Liu, Tong Li

**Affiliations:** 1 National Science Library, Chinese Academy of Sciences, Beijing 100190, China; songxf@mail.las.ac.cn; 2 Department of Information Resources Management, School of Economics and Management, University of Chinese Academy of Sciences, Beijing 100190, China; 3 Jiangsu Taizhou People’s Hospital, Taizhou 225306, China; docsong601@163.com; 4 School of Integrative Medicine, Nanjing University of Traditional Chinese Medicine, Nanjing 210023, China; 5 College of Life Sciences, University of Chinese Academy of Sciences, Beijing 100049, China; cuilizhen18@mails.ucas.ac.cn; 6 Albert Einstein College of Medicine and Montefiore Medical Center, Bronx, NY 10461, USA; tim.duong@einsteinmed.edu; 7 Indian Council of Forestry Research & Education, Dehradun 248006, India; rajivfri@yahoo.com; 8 Centre for Planetary Health and Food Security, School of Environment and Science, Griffith University, Nathan, Brisbane, QLD 4111, Australia; hongdou.liu@griffith.edu.au; 9 Department of Library, China Agricultural University (East Campus), 17 Qinghua East Road, Haidian District, Beijing 100193, China; qzhou@cau.edu.cn (Q.Z.); sy20235013757@cau.edu.cn (J.S.); 10 School of Agriculture and Food Sustainability, The University of Queensland, St. Lucia, Brisbane, QLD 4072, Australia

**Keywords:** coronavirus risk factors, multisystem impact, public health response, counter-measure

## Abstract

**Background:** Long COVID, characterized by a persistent symptom spectrum following SARS-CoV-2 infection, poses significant health, social, and economic challenges. This review aims to consolidate knowledge on its epidemiology, clinical features, and underlying mechanisms to guide global responses; **Methods:** We conducted a literature review, analyzing peer-reviewed articles and reports to gather comprehensive data on long COVID’s epidemiology, symptomatology, and management approaches; **Results:** Our analysis revealed a wide array of long COVID symptoms and risk factors, with notable demographic variability. The current understanding of its pathophysiology suggests a multifactorial origin yet remains partially understood. Emerging diagnostic criteria and potential therapeutic strategies were identified, highlighting advancements in long COVID management; **Conclusions:** This review highlights the multifaceted nature of long COVID, revealing a broad spectrum of symptoms, diverse risk factors, and the complex interplay of physiological mechanisms underpinning the condition. Long COVID symptoms and disorders will continue to weigh on healthcare systems in years to come. Addressing long COVID requires a holistic management strategy that integrates clinical care, social support, and policy initiatives. The findings underscore the need for increased international cooperation in research and health planning to address the complex challenges of long COVID. There is a call for continued refinement of diagnostic and treatment modalities, emphasizing a multidisciplinary approach to manage the ongoing and evolving impacts of the condition.

## 1. Introduction

The COVID-19 pandemic, caused by the SARS-CoV-2 virus, has been an unprecedented disruptor since its emergence in 2020, accruing an estimated 774.6 million confirmed cases globally [1]. The World Health Organization has identified five variants of the SARS-CoV-2 virus: Alpha (α), Beta (β), Gamma (γ), Delta (δ), and Omicron. These variants exhibit differences in transmissibility, disease progression, and severity, and may significantly impact the long-term health outcomes of affected individuals. These post-infection consequences are often referred to as Post-Acute Sequelae of SARS-CoV-2 infection (PASC) or long COVID, also known as long-haul COVID [2,3]. Long COVID significantly impacts physical, mental, and socio-economic aspects of patients’ lives. Studies have shown that the ongoing symptoms drastically reduce quality of life, leading to decreased work productivity, social isolation, and increased healthcare utilization [4,5,6,7]. Patients report chronic fatigue, pain, cognitive disturbances, and emotional distress, which profoundly affect daily living and well-being [4,5,6,7].

The term “long COVID” first emerged on social media in May 2020, becoming the inaugural “infodemic” to spread across Twitter and other media platforms. It describes the phenomenon where patients exhibit symptoms for weeks or months following their initial infection with the coronavirus [8]. Long COVID is a multi-systemic, potentially debilitating condition, raising concerns about permanent functional impairments [9]. In the autumn of the same year, the World Health Organization established the diagnosis for post-COVID-19 condition and assigned it the ICD-10 code U09 [10]. In October 2021, WHO members reached a consensus on the definition of post-COVID-19 condition using the Delphi technique. This allowed for the implementation of diagnostic codes in hospitals, enabling physicians to formally diagnose patients with long COVID and record these diagnoses in electronic health records [11]. Long COVID refers to individuals who continue to experience symptoms of COVID-19, persisting for at least two months and remaining unresolved three months after a suspected or confirmed infection, with no alternative diagnoses explaining these symptoms [12]. The definition of long COVID is evolving. For example, the Centers for Disease Control and Prevention (CDC) in the United States defines it as a range of new, recurring, or ongoing health problems that occur four weeks or more after the initial infection, including fatigue, fever, and various symptoms affecting the respiratory system, heart, nerves, and digestive tract [13]; the National Institute for Health and Care Excellence (NICE) in the UK describes it as persistent symptoms 4 to 12 weeks after the onset of acute symptoms, or as a post-COVID syndrome if symptoms persist beyond 12 weeks [14]; the Australian Department of Health states that symptoms persisting four weeks after the initial coronavirus infection constitute long COVID [15]. In Germany, 16 medical societies have compiled guidelines for long COVID and post-COVID, defining long COVID as symptoms that persist beyond four weeks post-infection or illness, and post-COVID as symptoms that are unexplained by other means and persist or emerge anew after 12 weeks [16].

At least 65 million people worldwide have had long-term infections of COVID-19 [4,8], with 23 million in the United States, 1.5 million in the United Kingdom, 1.4 million in Canada, 0.4 million in Australia, and over half of COVID-19 patients in Japan exhibiting symptoms of long COVID [9,10,11,12]. A case–control study conducted by Marra et al. (2023) among Brazilian healthcare workers revealed that up to 27% experienced long COVID after infection, with an increased risk of reinfection [13].

While the incidence and implications of long COVID are increasingly documented, significant gaps remain in understanding its long-term trajectory and management. This review aims to address these gaps by synthesizing the existing literature to evaluate the wide-ranging impact of long COVID and the efficacy of adaptive management strategies employed globally. It endeavors to provide a structured overview of the condition’s symptomatic patterns, risk factors, and the socio-economic repercussions faced by affected individuals and societies. By identifying key areas lacking robust evidence and highlighting regions where knowledge is fragmented or rapidly evolving, this study seeks to elucidate a coherent narrative of long COVID’s physiological mechanisms and its ripple effects across healthcare systems. Ultimately, this review intends to offer concrete insights for clinicians, researchers, and policymakers to refine approaches towards diagnosing, treating, and mitigating the multi-dimensional impacts of long COVID.

## 2. Materials and Methods

**Figure 1:**
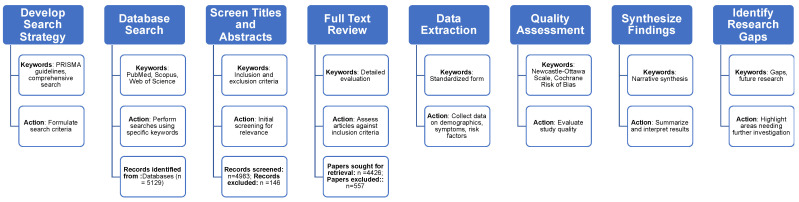
The framework of long COVID comprehensive review work.

In our review, we adapted to the Preferred Reporting Items for Reviews and Meta-Analyses (PRISMA) guidelines (http://www.prisma-statement.org/ (accessed on 23 April 2024)). We conducted a comprehensive search across multiple databases including PubMed, Scopus, Web of Science, and Embase from inception until December 2023, using a combination of keywords and MeSH terms related to “long COVID”, “Post-Acute Sequelae of SARS-CoV-2 infection (PASC)”, and “chronic COVID syndrome”. The literature scope included peer-reviewed articles, reviews, and gray literature that reported on the epidemiology, clinical manifestations, risk factors, and management strategies of long COVID. We excluded studies focused solely on the acute phase of COVID-19, those lacking specific data on long COVID, or non-English publications. Two independent reviewers screened titles and abstracts for eligibility. To enhance the objectivity and reliability of our quality assessment, any discrepancies between reviewers were initially discussed between them to reach a consensus. If disagreements persisted, a third reviewer was consulted to make a final decision. This triage approach helped reduce subjective bias in study selection and quality evaluation (Figure 1).

Data extraction was performed using a standardized form to capture information on study design, participant demographics, reported symptoms, risk factors, diagnostic criteria, therapeutic interventions, and outcomes. Quality assessment of included studies was conducted using the Newcastle-Ottawa Scale for observational studies and the Cochrane Risk of Bias tool for randomized controlled trials. The extracted data were synthesized narratively to capture the heterogeneity of the studies. The findings were organized thematically based on symptomatology, risk factors, pathophysiological hypotheses, and management strategies. Meta-analytic methods were not employed due to the anticipated variability in study designs and outcomes. This approach enabled a comprehensive summary of the current state of knowledge and identification of gaps in research on long COVID.

## 3. Results

### 3.1. Symptoms of Long COVID

**Figure 2:**
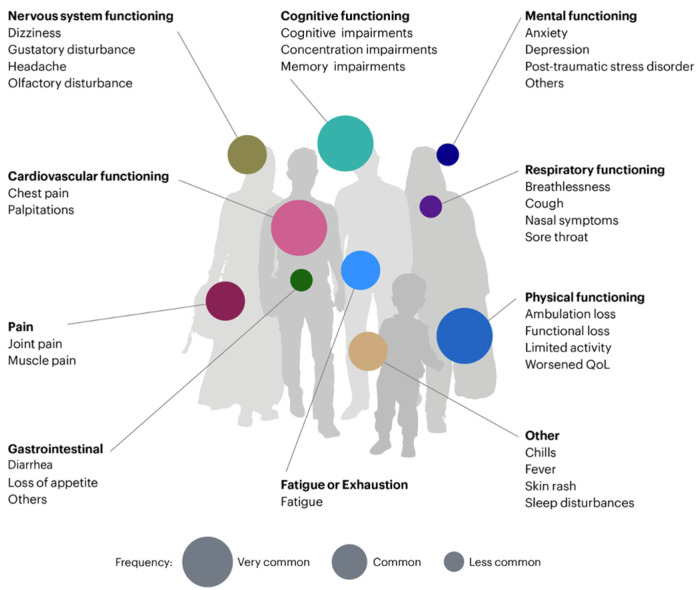
Some common symptoms of long COVID. Over 200 symptoms of long COVID have been reported in many different studies (adapted from: https://www.design-science.org.uk/news/visualising-long-covid, accessed on 1 May 2020).

Following the implementation of diagnostic measures, researchers have leveraged clinical data to discern the characteristics of long COVID. The researchers tracked 3762 confirmed or suspected patients from 52 countries, all with illness durations exceeding 28 days, identifying 203 long COVID symptoms related to 10 human organ systems [14], as shown in Figure 2.

#### 3.1.1. Typical Symptoms of Long COVID

Typical symptoms of long COVID include fatigue, headache, body aches, shortness of breath, cognitive issues (such as brain fog), chest pain, and palpitations, with varying prevalence and severity [7,15,16]. Gutierrez-Martinez (2022) conducted a retrospective data analysis from February 2020 to May 2021, including patients with persistent symptoms after short-term hospitalization or outpatient patients who were never hospitalized and observed that individuals with brain fog exhibited a range of issues including insufficient sleep, anxiety, depression, reduced attention, and executive function impairments [17]. Long COVID patients frequently experience autonomic dysfunction and Postural Orthostatic Tachycardia Syndrome (POTS) [18,19]. Headaches, anxiety, and depression are more prevalent among younger patients and females, while older patients are more likely to suffer from cognitive deficits and respiratory issues, and males are more prone to muscle or joint pain [20]. Additionally, loss of smell and taste, insomnia, and gastrointestinal problems also occur alongside the primary symptoms [15,16].

#### 3.1.2. Duration of Long COVID in the Patient

Dennis (2023) found that 59% of long COVID patients continued to exhibit organ damage one year after the initial symptoms, and 39% experienced persistent pulmonary function impairment two years later [21]. Baskett et al. (2022) studied the 47 most common symptoms of long COVID and found that only seven symptoms, including accelerated heart rate, hair loss, fatigue, chest pain, shortness of breath, joint pain, and obesity, are manifest within the first year post-infection [22]. Jia et al. (2022) determined that the median time to initial symptom resolution is 44 days post-diagnosis, with a median duration of 214 days to resolve all symptoms [23]. Symptoms of COVID-19 persisting beyond 15 weeks may last for a year.

### 3.2. Risk Factors and Physiological Mechanisms That Trigger Long COVID

**Figure 3:**
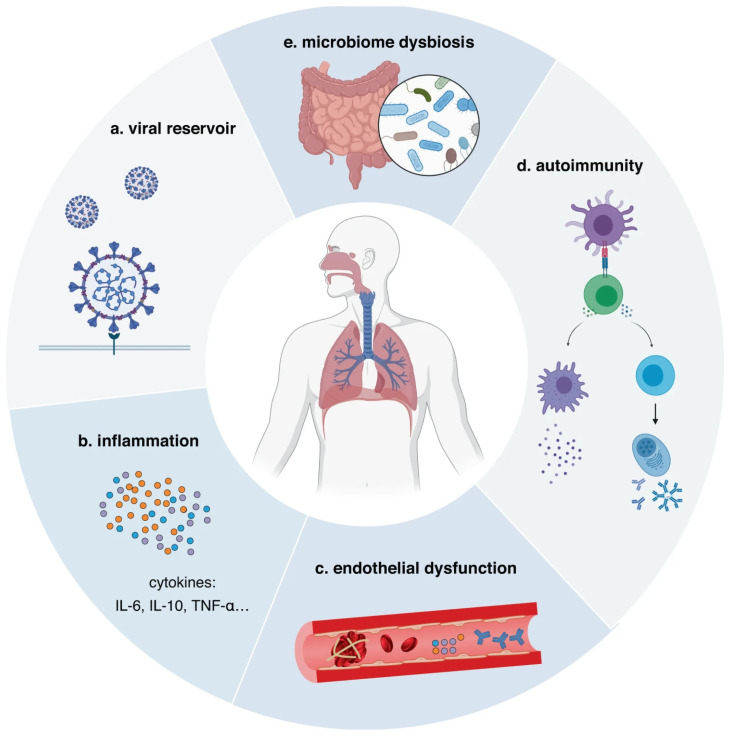
The potential pathophysiological mechanisms of long COVID (adapted from: https://www.nature.com/articles/s41392-023-01640-z, accessed on 1 May 2020).

Davis et al. have posited various hypotheses regarding the pathogenesis of long COVID, including immune dysregulation, dysbiosis of the microbiome, autoimmunity, coagulation and endothelial abnormalities, and neurological dysfunction [4]. Given the widespread and nonspecific nature of long COVID, comprehending the associated risk factors and physiological mechanisms is crucial for clinicians and healthcare organizations in proficiently screening, monitoring, and treating patients (Figure 3). Furthermore, this information empowers individuals by providing them with insights into their medical history, enabling a comprehensive assessment of the risk associated with long-term COVID-19 infection for enhanced disease prevention [24,25,26,27].

#### 3.2.1. Risk Factors for Long COVID

(1) Gender, age, and pre-existing chronic conditions emerge as pivotal risk factors influencing the susceptibility to long COVID. Notably, the risk of long COVID in women is twice that of men [28]. Age-wise, individuals aged 60 to 79 face a 25% elevated risk compared to their younger people, escalating to a 60% higher risk for those over 80. Distinct patterns also emerge in the context of hospitalization. Patients requiring hospitalization for COVID-19 are at a substantially greater risk of developing long COVID, with the risk escalating up to 18 times. Remarkably, those necessitating invasive ventilation, such as mechanical ventilation, face an exponentially increased risk of 115 times [29]. Pre-existing health conditions play a pivotal role in long COVID diagnoses. Patients with a history of cardiovascular diseases, chronic lung diseases, kidney diseases, hypertension, diabetes, obesity, and depression before contracting COVID-19 are more predisposed to long COVID [30]. Additionally, individuals with rheumatic diseases, contracting the coronavirus and experiencing a heightened antibody response that triggers an immune reaction, are notably more prone to developing long COVID [31]. These nuanced risk factors provide critical insights for a comprehensive understanding of long COVID.

(2) Smoking, high body mass index, life stress, and psychological factors emerge as additional risk factors contributing to the complexity of long COVID. In a six-month follow-up study of discharged COVID-19 patients, researchers observed a significant correlation between high muscle atrophy and increased fatigue and muscle pain, highlighting the nuanced impact of physical health on long COVID outcomes [32]. A noteworthy finding indicates that women maintaining a healthy lifestyle—marked by factors such as a balanced weight, non-smoking status, regular exercise, sufficient sleep, a high-quality diet, and moderate alcohol consumption—experience a 49% reduced risk of long COVID compared to those with unhealthy lifestyles [33]. Furthermore, a robust association exists between the severity of long COVID and the initial severity of the COVID-19 infection [34]. Additionally, major life stressors, encompassing economic challenges, food security issues, and death of close contacts, have been identified as contributors to an increased risk of long COVID [35]. Psychological factors also play a pivotal role, with pre-existing distress, including towards anxiety, worry, perceived stress, and loneliness, leading to an elevated long COVID risk ranging from 32% to 46%. Additionally, these psychological factors contribute to a heightened risk of impaired daily functioning, ranging from 15% to 51% [33]. This comprehensive exploration sheds light on the intricate interplay between various factors and their impact on long COVID.

#### 3.2.2. Physiological Mechanisms of Long COVID Formation

The occurrence of long COVID is linked to the formation of a viral reservoir within the body subsequent to the initial infection. It triggers an overly active immune system, characterized by CD^8+^ T-cell counts that can surge up to 100 times higher than normal, thereby dampening overall immune response [36,37,38]. Concurrently, diminished levels of anti-nuclear envelope antibodies and the presence of pulmonary diseases contribute to the manifestation of long COVID symptoms [23,32]. Furthermore, COVID-19 infection induces immune dysfunction, resulting in nerve damage such as dysfunction of the vagus nerve and a reduction in olfactory sensory neurons, which in turn induce long COVID symptoms [39,40,41,42,43]. Significant differences between the circulating antibodies and other components of the immune system are noted in long COVID patients compared to other patient groups [44]. Long COVID patients have notably lower cortisol levels, aiding in the development of related biomarkers and diagnostic and treatment methods [35,44]. Researchers, utilizing a real-time deformability cytometer (RT-DC) they developed, discovered that changes in blood cells lead to long COVID, with damaged blood cells increasing the risk of vascular blockages and pulmonary embolism [45]. Carlo (2024) identified changes in serum proteins of long COVID patients, including the activation of the immune system’s complement cascade, coagulation changes, and tissue damage, providing potential biomarkers for the diagnosis of long COVID [46]. Greene (2024) revealed that the integrity of brain blood vessels in long COVID patients is compromised and the leaking vessels, coupled with an overactive immune system, are a key driver of long COVID-related brain fog, assisting in the development of targeted treatments for patients [47].

### 3.3. The Social Economic Impact of Long COVID

Long COVID imposes restrictions on patients’ daily activities, severely affects their ability to return to work or social life, subsequently impacting their psychological health, and causes significant economic loss to patients, their families, and society [48,49,50].

#### 3.3.1. The Impact of Long COVID on Society

Previously, a national survey in the UK found that patients treated for COVID-19 in hospitals had still not fully recovered after five months, and had limited health recovery after one year, with fewer than 3 out of 10 patients fully recovering [51]. Non-hospitalized patients in France experienced long COVID symptoms for up to two years, with a minority losing functional capacity for as long as 22 months [52]. Aburto (2022) surveyed 29 countries that had COVID-19 outbreaks and found that life expectancy decreased by more than 1 year documented in 11 countries for males and 8 for females, and males experienced with the largest decreases in life expectancy for 2.2 years in the USA and 1.7 years in Lithuania [53]. Severe COVID-19 has a lasting effect on memory, attention, and problem-solving abilities equivalent to aging 20 years in patients, causing persistent cognitive impairments in 50- to 70-year-old patients equivalent to a 10-point reduction in IQ [54]. From surveying 1000 Wuhan residents infected before December 2020, it was found that more than half of the COVID-19-recovered had post-COVID conditions after infection of 20 months, with nearly 1/6 suffering from severe sequelae [55]. Cognitive effects and fatigue are the main causes for the patients to return to work, with cognitive symptoms unrelated to outward physical disability [56].

#### 3.3.2. Long COVID’s Impact on the Economy

As of June 2022, data from the U.S. National Bureau of Statistics reported that over 16 million working-age Americans (aged 18 to 65) were infected by long COVID, with approximately 4 million people losing their jobs due to the disease. The resultant loss of jobs equates to an annual loss of USD 170 billion in wages, nearly 1% of the total U.S. gross domestic product [57]. On 5 December 2022, data from the UK’s Office for National Statistics showed that nearly 2 million people were experiencing long COVID, with their economic inactivity rate being close to ten times that of healthy individuals. A Bank of England survey found that labor force participation among long COVID sufferers aged 16 to 64 fell by 1.3%, and a quarter of UK companies cited long COVID as one of the main reasons for staff absenteeism [58]. There are many other countries also put a lot of efforts and struggle for long COVID [59,60,61,62,63,64,65,66,67]. Davis et al. (2021) found that about 20% of long COVID patients were out of work, and nearly half had reduced their working hours [14]. Although there is limited evidence of acute or long-term COVID-19 infections among the Japanese population, about 70% of COVID-19 patients in Japan believe that post-COVID conditions affect their work, such as unemployment or suspension [68]. Additionally, data indicate that, as of 30 September 2022, approximately 1.2% of COVID-19-related deaths in Australia were due to long COVID, and the burden of long COVID accounts for about 10% of the total COVID-19 burden in Australia. Patients with long COVID experience a significant decline in their ability to perform daily activities, with persistent symptoms affecting labor participation and delaying return to work [69].

#### 3.3.3. New Clinical Disorders and Accelerated Disease Progression

Individuals who survive acute COVID-19 may be at higher risk of developing new clinical disorders and/or accelerated disease progression of existing disorders. Higher incidences of diabetes [70,71,72], hypertension [73], and kidney disorders [74,75], among others, have been reported in individuals post-COVID-19 compared to non-COVID matched controls. Worsening of disease progression of existing clinical disorders has been reported in patients with hypertension [76,77], kidney disease [78], multiple sclerosis [79,80,81,82,83,84], dementia [85,86,87], and other neurological conditions [86,87,88,89,90,91] post-COVID-19 compared to non-COVID matched controls. We expect that there will be accelerated aging of multiorgan systems in some individuals, especially those with experience of severe acute COVID-19 and/or with major pre-existing comorbidities. These new-onset disorders and accelerated disease progression are expected to result in increased healthcare burden in years to come. Identifying at-risk individuals and risk factors may encourage additional health monitoring post-SARS-CoV-2 infection.

### 3.4. Reponses of Major Countries to Long COVID

#### 3.4.1. Reducing the Impact of Long COVID on the Workforce through Policy Adjustments

Policy measures include ensuring better prevention and treatment options, expanding paid sick leave, improving workplace accommodations, and increasing access to disability insurance. The Americans with Disabilities Act stipulates that long COVID patients are eligible to be classified as disabled and enables to receive appropriate healthcare to regain productivity [59,92]. The UK’s Equality Act considers all long COVID sufferers as disabled, entitled to support and formal protection under employment law necessary for continued work, and long COVID are recognized as an occupational disease, with entitlement to protection and compensation for infections contracted during employment [60,93].

The establishment of long COVID clinics provides patients with diagnosis, treatment, and care. As of January 2023, 477 hospitals in Tokyo, Japan, had set up clinics for post-COVID-19 sequelae. Italy offers convenience to long COVID patients through a community care network, with non-profit organizations providing remote monitoring systems, expert intervention, psychological support, and services such as neurological, respiratory, and cognitive rehabilitation post-hospitalization. Australia has also opened long COVID specialty clinics in the capital and regional centers, providing additional care for patients [61,94]. The University of Pennsylvania’s School of Medicine has established a COVID Aftercare and Recovery Clinic, offering multidisciplinary assessment and treatment resources for patients recovering from COVID-19, ensuring continuity of patient care, and reducing long-term morbidity and mortality [13,62]. Hospitals in Beijing, Guangzhou, Hangzhou, Shanghai, Wuhan, Zhengzhou, and other places have already set up post-COVID infection recovery clinics or specialist joint clinics, providing patients with comprehensive diagnosis and treatment, including condition assessment, functional training, traditional Chinese medicine treatment, and psychological counseling [63,95].

#### 3.4.2. By Offering Government Fundings to Support Foundational Research on Long COVID

Since 2020, the Canadian Institutes of Health Research has funded USD 17.7 million for targeted research on post-COVID-19 sequelae. The Canadian government provided CAD 20 million for the establishment of the Canadian COVID-19 Sequelae Research Network at the beginning of 2023, to study the biological basis of the disease and its impact on clinical care, mental health, health systems, and population health, in order to formulate related plans and policies [64,96]. In 2021, the German statutory pension insurance and German social accident insurance allocated EUR 332 million for health research to combat post-COVID sequelae, developing treatments for outpatient care or rehabilitation [65,97]. In 2021, the UK Research and Innovation (UKRI) and the National Institute for Health Research (NIHR) jointly funded £18.5 million for the Post-HOSPitalisation COVID-19 study (PHOSP-COVID) to identify the causes of long COVID and effective treatments for patients with chronic disease symptoms [66,98]. As of May 2022, the UK government invested over GBP 50 million in government funds to research the potential mechanisms of long COVID and symptoms such as brain fog and respiratory difficulty, testing possible treatments. In March 2022, the Japan Society for the Promotion of Science funded JPY 300 million, and the UKRI contributed GBP 3.5 million to establish 10 collaborative research projects, focusing on the global impact of long COVID [67,99]. In February 2022, the U.S. National Institutes of Health invested USD 470 million to support large-scale studies on the impact of long COVID [90,100]. In August 2022, the White House released a national research action plan on long COVID, allocating USD 1.15 billion for research into the symptoms of long COVID and how the virus triggers these symptoms [101]. In the USA, there are two major long-COVID initiatives funded by the NIH [102,103].

#### 3.4.3. Conducting Investigation to Develop Effective Treatment Strategies for Long COVID

The over-elimination of synapses by brain immune cells aids in identifying new treatments for persistent cognitive symptoms after coronavirus infections [94,104]. Treatment of post-COVID-19 sequelae with autologous fat-derived mesenchymal stem cells is first implemented in Japan, producing positive outcomes through anti-inflammatory actions and tissue regeneration [95,96].

Mucosal antibodies can provide lasting protection against coronavirus infections. Havervall (2022) found that respiratory persistent mucosal antibodies against infection with Omicron variants BA.1 or BA.2 offer durable protection, which reduce the risk of reinfection with BA.5 by 90% [97]. Yan (2023) showed that intranasal administration of platelet-rich plasma extracted from the patient’s own blood treat taste loss related to long COVID [98].

Therapies involving certain components may be effective for certain symptomatologies. Rosemary contains carnosic acid (CA) and carnosol (CS), abietane-type phenolic diterpenes, which account for most of its biological and pharmacological actions, however, CA-related compounds can serve as drugs for treating coronavirus infection-related brain sequelae, penetrating the blood–brain barrier and reaching the brain parenchyma to protect nerves [97,105]. Colchicine is promising for treating long COVID, potentially reducing complications associated with long COVID [98,104]. Lithium has anti-inflammatory and neuroprotective properties, and low-dose lithium aid in the recovery of long COVID in adults [99,100]. Many effective treatments and rehabilitation methods for functional disorders are also being used in the diagnosis and treatment of long COVID [105].

Advancing the development of monitoring methods for long COVID. Fagherazzi (2022) innovatively used various devices and vocal recordings of breathing to identify COVID-related symptoms, allowing for long-term monitoring of long COVID patients and predicting the evolution of their symptoms [106]. Kovarik (2023) utilized mass spectrometry-based post-genomic analysis techniques to identify symptoms associated with long COVID [107]. Research by Kuchler (2023) et al. demonstrated that ocular blood vessels change due to persistent coronavirus symptoms; standardized ophthalmological examinations reveal whether individuals are suffering from long COVID syndrome in the future [108].

#### 3.4.4. Conducting Development of Vaccines in Prevention and Protection against Long COVID

Between August 2021 and June 2022, following the rollout of vaccines, referrals to the long COVID clinic in Cambridge, UK, sharply decreased by 79% [109]. Al-Aly’s (2022) analysis of patients and controls in the U.S. Department of Veterans Affairs health care database showed that vaccination reduced the risk of long COVID by 15%, though this study did not include data on individuals infected with the Omicron variant [110]. Mizrahi (2023) et al. conducted a retrospective analysis of electronic medical records from the Israeli national health institution and found that vaccination reduced the risk of persistent breathing difficulties in patients with breakthrough infections [11,20]. Richard’s (2023) research indicates that vaccination reduces the risk of persistent symptoms [111]. Marra’s (2023) conducted a meta-analysis of the literature in databases from December 2019 to June 2023 and showed that the effectiveness of three vaccine doses against long COVID is 69%, while two doses is 37% effective; vaccination prior to COVID-19 infection significantly reduced long COVID cases [13]. The next-generation vaccine GRT-R910, developed by biotechnology company Gritstone using self-amplifying mRNA (samRNA), induces a strong and lasting immune response, potentially providing broad and long-term clinical protection [112].

## 4. Discussion

### 4.1. Comparison with Other Infections

COVID-19 and influenza share similarities as both viruses primarily affect the lungs and cause symptoms of upper respiratory infection, such as coughing, runny nose, sore throat, fever, headache, and fatigue. Both can be deadly and are easily transmitted through the respiratory tract [113]. However, COVID-19 is associated with a higher risk of death compared to influenza. Patients with COVID-19 are more likely to be obese or overweight, and more often suffer from diabetes, high blood pressure, and abnormal blood lipids, whereas influenza patients more frequently experience heart failure, chronic respiratory diseases, cirrhosis, and deficiency anemia [114]. Compared to those with influenza, COVID-19 hospitalized patients more frequently suffer from acute respiratory failure, pulmonary embolism, septic shock, or hemorrhagic stroke, but have lower incidences of myocardial infarction or atrial fibrillation [115]. Therefore, responding to COVID-19 variants and improving prevention and treatment strategies remain highly relevant.

### 4.2. Future Adaptive Management Suggestions and Directions

**Figure 4:**
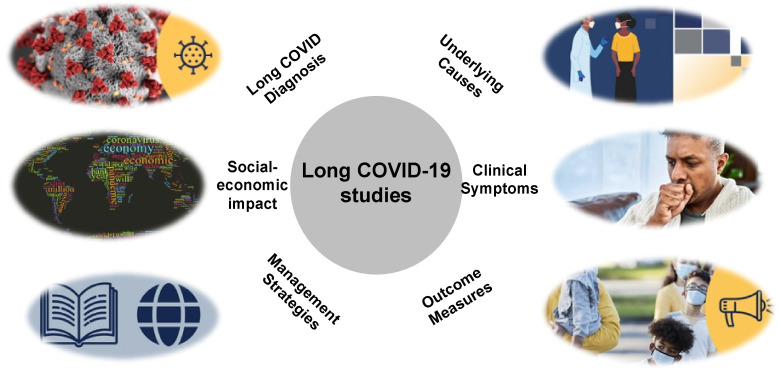
The framework of long COVID management.

Despite the expansion and acceleration of global research on long COVID, there remain significant challenges, and existing studies are not sufficient to fully improve the prognosis of long COVID patients. There is also the need to address future variants of the SARS-CoV-2 virus (Figure 4).

(1) Intensify research on the mechanisms of long COVID and devise effective medical plans. The plan may involve screening for effective biomarkers to assess and predict the course of long COVID. Developing effective diagnostic and imaging tools may also be used for the safe diagnosis of long COVID. Unravelling the possible physiological mechanisms for the long COVID symptoms, performing large-scale clinical trials and discovering effective treatment methods could help improve the health and quality of life of long COVID patients. These plans may face challenges such as funding limitations, variability in biomarker effectiveness across diverse populations, and potential delays in clinical trial outcomes. To mitigate these issues, we recommend prioritizing funding for high-impact research areas, using adaptive clinical trial designs, and promoting international collaborations to increase the diversity of clinical trial participants [115,116].

(2) Within the bounds of the law, enhance multi-institutional data sharing on long COVID, implement stratified management of patients, provide effective treatments, and promote a path for full recovery. While enhancing data sharing and stratified patient management is pivotal, concerns about data privacy, interoperability of health information systems, and unequal access to care must be considered [117,118,119]. Strengthening data protection laws, investing in technology to enhance system compatibility, and establishing clear guidelines for equitable patient management can address these limitations.

(3) Enhance the training and education of healthcare and research personnel, as well as public awareness, which may also support minimizing the risk of long COVID. Improve education about pandemics, viruses, and infection-induced diseases (such as long COVID), which may ensure that people with long-term coronavirus and related diseases receive adequate care, and implement effective interventions to prevent worsening prognoses. Educational initiatives might not reach all segments of the population equally, and misinformation could undermine public awareness efforts [120]. To overcome these barriers, tailored educational programs that cater to different demographic groups and robust fact-checking mechanisms are essential. Collaborations with trusted community leaders and influencers can also enhance the reach and impact of educational campaigns.

(4) Increase policy interventions to reduce the socio-economic impact of long COVID. Conduct long COVID infection surveillance and analysis of its long-term impact on the workforce, healthcare costs, and societal and economic aspects, and develop corresponding policies and guidelines. This would also be useful. Optimize the organization of medical and healthcare services to ensure every patient has timely access to evidence-based and cost-effective treatment. Through population surveys, count individuals unable to work due to long COVID and establish occupational rehabilitation plans to support their re-employment and provide labor protection. Policy interventions intended to mitigate the socio-economic impacts of long COVID require careful consideration to avoid unintended consequences, such as stigmatization or resource allocation biases. Continuous monitoring of policy outcomes, stakeholder engagement, and flexible policy frameworks that can adapt to new evidence and changing circumstances are recommended [121]. By proactively addressing these potential limitations and adapting our strategies accordingly, we can enhance the resilience and effectiveness of our management approaches, ultimately improving outcomes for individuals affected by long COVID and society at large.

### 4.3. Limitation of This Research

The limitations of this review are primarily associated with the scope of the literature analyzed and methodological constraints. Despite employing a comprehensive search strategy across multiple databases, the review was restricted to articles published in English, potentially overlooking pertinent studies in other languages that could influence the findings. Moreover, the exclusion of grey literature and unpublished studies might have introduced publication bias, favoring studies with significant results. The quality and heterogeneity of the included studies also presented challenges. Variations in study design, methodology, and quality across the papers could impact the consistency and generalizability of the review’s conclusions. Additionally, due to the variability in expected outcomes, meta-analytic methods were not utilized, which may limit the capacity to quantitatively assess the effectiveness of different management strategies. Finally, the continual evolution of research on long COVID suggests that new information might emerge that was not available at the time of this review, indicating the need for ongoing research to constantly update our understanding of the condition.

## 5. Conclusions

This comprehensive review elucidates the multifaceted nature of long COVID, revealing a broad spectrum of symptoms, diverse risk factors, and the complex interplay of physiological mechanisms underpinning the condition. The global impact of long COVID extends beyond the medical to significant socio-economic ramifications, underscoring the need for a multidisciplinary approach to management and intervention. The emergence of diagnostic criteria and therapeutic strategies marks a significant advancement in our understanding and ability to address long COVID. However, the variability in symptomatology, coupled with the evolving nature of SARS-CoV-2 and its variants, highlights the imperative for ongoing research.

The international response to long COVID, while varied, demonstrates a concerted effort to mitigate the condition’s impact through clinical interventions, policy adjustments, and community support mechanisms. Nevertheless, this review identifies critical gaps in long-term management strategies and the necessity for adaptive approaches to accommodate the dynamic and persistent challenges posed by long COVID. One of the challenges is to distinguish whether these symptoms, new disorders, and disease worsening are truly due to COVID-19 disease or are part of natural aging or have been undiagnosed. Careful studies are needed. Future endeavors should focus on unraveling the intricate pathophysiological pathways of long COVID, developing precise diagnostic tools, and tailoring patient-centric therapeutic interventions. Furthermore, enhancing data sharing, strengthening healthcare infrastructure, and fostering public awareness are pivotal in curbing the spread and impact of long COVID. Policymakers and healthcare providers must collaborate to devise robust frameworks that address the socio-economic aspects of long COVID, ensuring comprehensive support for affected individuals and communities.

In conclusion, while significant strides have been made in understanding and managing long COVID, the journey towards fully addressing this global health challenge is ongoing. Multidisciplinary research, international cooperation, and innovative management strategies are essential in navigating the complexities of long COVID and enhancing the quality of life for those affected.

## Data Availability

Not applicable.
